# Supplementary material for: Targeting mosquito X-chromosomes reveals complex transmission dynamics of sex ratio distorting gene drives
Source: Nat Commun. 2024 Jun 11;15:4983. doi: 10.1038/s41467-024-49387-7 (PMC11166636; doi:10.1038/s41467-024-49387-7)
Supplement: Supplementary file 1 — Supplementary Information [file 41467_2024_49387_MOESM1_ESM.pdf]

Table S1. Primer and oligo sequences utilized for plasmid construction.

Constructs and primers	Primer sequence (5' to 3')	Source
952_2 sgRNA		
952_2 F	AAATGGAGTTGCTCTCTGCTAGGACGATCTACCACAACGGGTTTTAGAGCTAGAAATAGC	Self-annealing primers
952_2 R	GCTATTTCTAGCTCTAAAACCCGTTGTGGTAGATCGTCCTAGCAGAGAGCAACTCCATTT	
953_1 sgRNA		
953_1 F	AAATGGAGTTGCTCTCTGCTGAGCCTACAGGGTATAACATGTTTTAGAGCTAGAAATAGC	Self-annealing primers
953_1 R	GCTATTTCTAGCTCTAAAACCCGTTGTGGTAGATCGTCCTAGCAGAGAGCAACTCCATTT	
739_1 sgRNA		
739_1 F	ATGGAGTTGCTCTCTGCTGCCGACAAGAAGGGAGACGT	Self-annealing primers
739_1 R	GCCGACAAGAAGGGAGACGTGTTTTAGAGCTAGAAATA	
Xshrd;952_1		
Xshrd,952_1F	aaatggagttgctctctgctGGGTACAAGCTTGCGTACGT	Gene synthesized vector
Xshrd,952_1R	gctatttctagctctaaaacCCGGTACCGGTCGTATGCGC	
952_1;952_2		
952_1,952_2F	aaatggagttgctctctgCTGCGCATACGACCGGTACC	Gene synthesized vector
952_1,952_2R	gctatttctagctctaaaacCCGTTGTGGTAGATCGTCCT	
An. gambiae zpg promoter		
zpg F	tcttcctactgcaggaaccggtaggcgcgcccaGCGCTGGCGGTGGGGA	A. gambiae genomic DNA
zpg R	cttatagtccatctcgatgctgtaTTTGTTGTTGGGCTGCTTG	
An. gambiae zpg 3'UTR		
zpg 3'UTR_F	cggccaggcaaaaaagaaaaagGAGGACGGCGAGAAGTAATC	A. gambiae genomic DNA
zpg 3'UTR_R	ttaaactttaattaatacgcgttggcgcgcccATAATGAACGAACCAAAGGG	
D. melanogaster βtub85D promoter		
βtub85D_ F	cacggtgagcacgggacgtgcgacggtcaatATCAATCGTATCATCTGGTCG	*D. melanogaster* genomic DNA
βtub85D_R	tccgtcgtggtccttatagtccatccattttGATAGTAAAGTTAGGGCCCC	

D. melanogaster βtub56D 3'UTR		
βtub56D_ F	atggacgagctgtacaagtaaTGAGGTCGACGAGAACTAAATTC	*D. melanogaster* genomic DNA
βtub56D_R	ggaggccaccgagtatggGTCATGTCCTCTTCAAGGGCGAA	
D. melanogaster RpS6_2 sgRNA		
RpS6_2_F	TATATATAGGAAAGATATCCGGGTGAACTTCGCCGGCGGCAACGACAAGCAGTTTTAGAGCTAGAAATAGCAAG	Self-annealing primers
RpS6_2_R	CTTGCTATTTCTAGCTCTAAAACTGCTTGTCGTTGCCGCCGGCGAAGTTCACCCGGATATCTTTCCTATATATA	

*Gibson homology arms appear in undercase.

Table S2. Primers utilized for amplicon sequencing.

Target	sgRNA name	sgRNA sequence	Forward primer	Reverse primer	Amplicon size (bp)
AGAP000739	739_1	5'-GCCGACAAGAAGGGAGACGTCGG-3'	5'-acactgacgacatggttctacaCATCTGCCGTCCGAGATCG-3'	5'-tacggtagcagagacttggtctCGAACTTGAAAAACACCCGGA-3'	411
AGAP000952	952_1	5'-GCGCATACGACCGGTACCGGTGG-3'	5'-acactgacgacatggttctacaACGACAGCTCCTTGCAATCA-3'	5'-tacggtagcagagacttggtctACTGCTTAAGCTGCGGACTT-3'	415
AGAP000952	952_2	5'-AGGACGATCTACCACAACGGCGG-3'	5'-acactgacgacatggttctacaGCATCCTGCGACTCGTTC-3'	5'-tacggtagcagagacttggtctGAAAGAAAATACAAGGGCATCTGGC-3'	413
AGAP000953	953_1	5'-GAGCCTACAGGGTATAACATAGG-3'	5'-acactgacgacatggttctacaAGCCACGTGTCTGCAAC-3'	5'-tacggtagcagagacttggtctTTACGCGGAGAGTCGATTGC-3'	465
28S rDNA	Xshrd	5'-GGGTACAAGCTTGCGTACGTCGG-3'	5'-acactgacgacatggttctacaCATGATTGGGTGGAACAAGCG-3'	5'-tacggtagcagagacttggtctGACCTTTGGACACCTCCGTT-3'	472

*PAM sites are underlined.

**Illumina adapters appear in undercase.

Table S3. Single embryo genotyping – PCR success rates.

Cross	Original sample size	Successful reactions	Success rate
WT males x WT females	95	75	78.95
739_1_c males x WT females	100	91	91.00
952_1;952_2_a males x WT females	100	96	96.00
952_1;Xshrd males x WT females	119	90	75.63

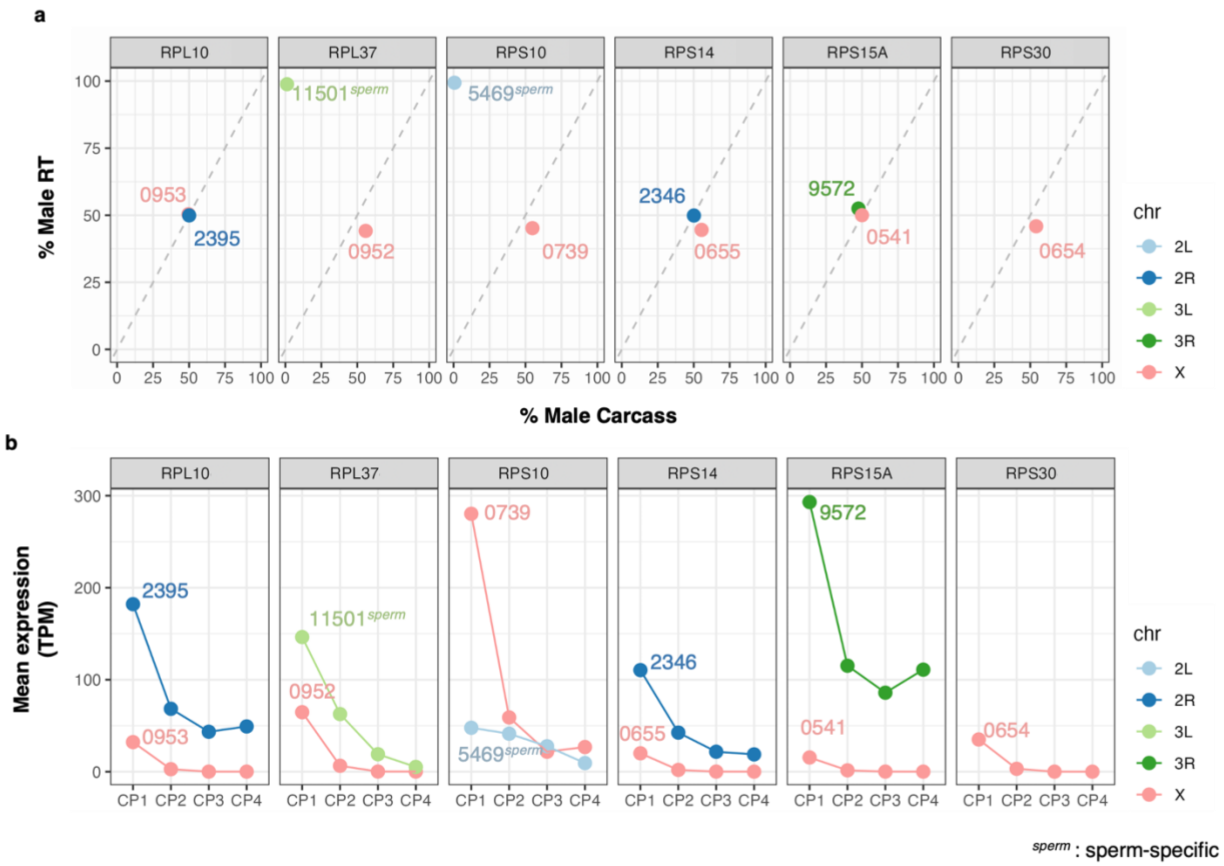

Figure S1. Transcriptional dynamics of X-linked ribosomal proteins genes and autosomal paralogs. (a) For each ribosomal gene, normalized expression in the male carcass and in male reproductive tissues (RT), obtained from Papa et al. (2017)[1], was downloaded from *Vectorbase.org* and plotted as a percentage of the sum of expression of both tissues combined. The functional name of each ribosomal protein gene is indicated in the corresponding facet subtitle. Gene IDs are shown within the plot (omitting "AGAP00") and colored according to chromosomal arm. The diagonal intercept represents equal expression in somatic and germline male tissues. Genes labeled "sperm" are expressed exclusively in sperm according to data from additional tissues. **(b)** For each ribosomal gene, normalized mean expression is shown (in Transcripts Per Million, TPM) at four stages of sperm development collected by FACS sorting in cell populations (CP); premeiotic (CP1), early (CP2) & late meiotic (CP3) and postmeiotic (CP4)

sperm cells. RNA-seq data for this analysis was obtained from Taxiarchi et al. (2019)[2] from *Vectorbase.org*.

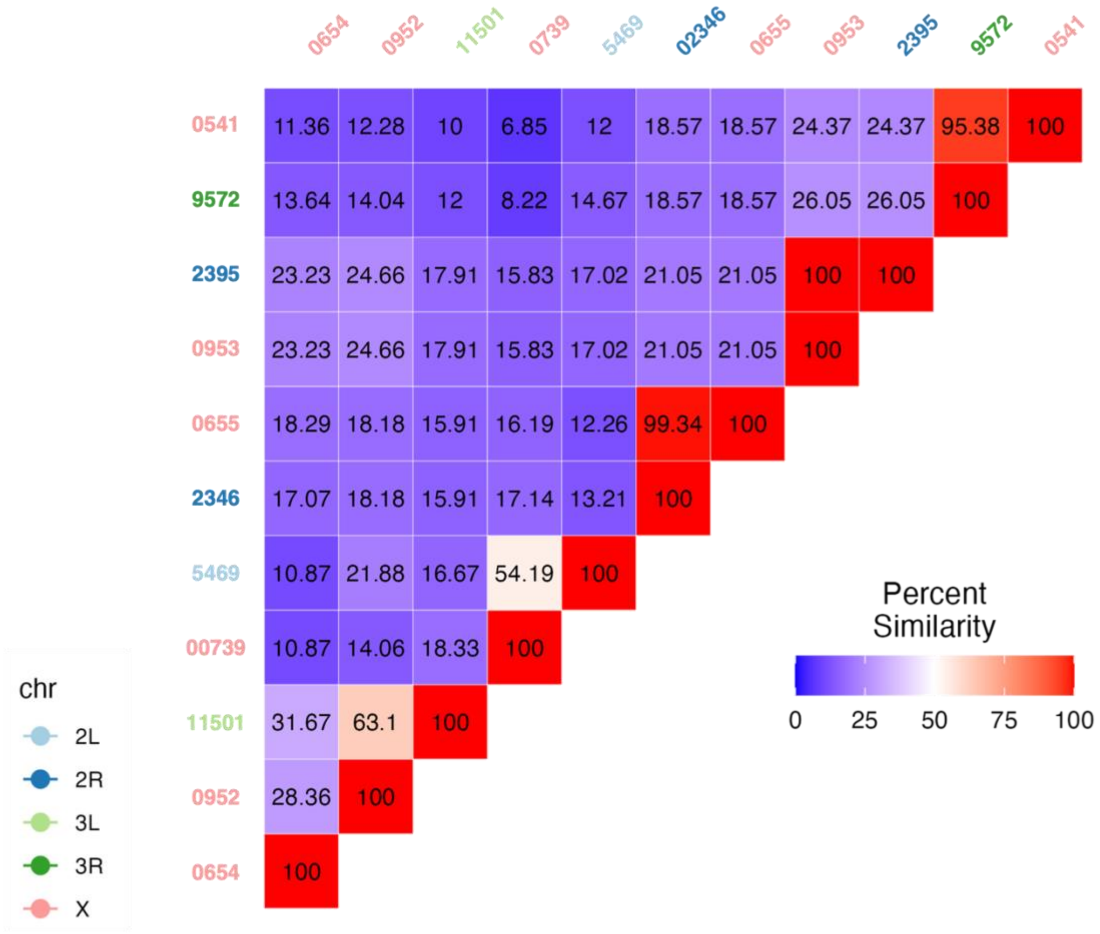

Figure S2. Similarity of *An. gambiae* X-chromosome ribosomal proteins and their autosomal paralogs. Protein sequences for each gene were downloaded from *Vectorbase.org*. Similarity (shown as % identity) matrix of mosquito ribosomal protein genes based on Clustal-Omega alignment.

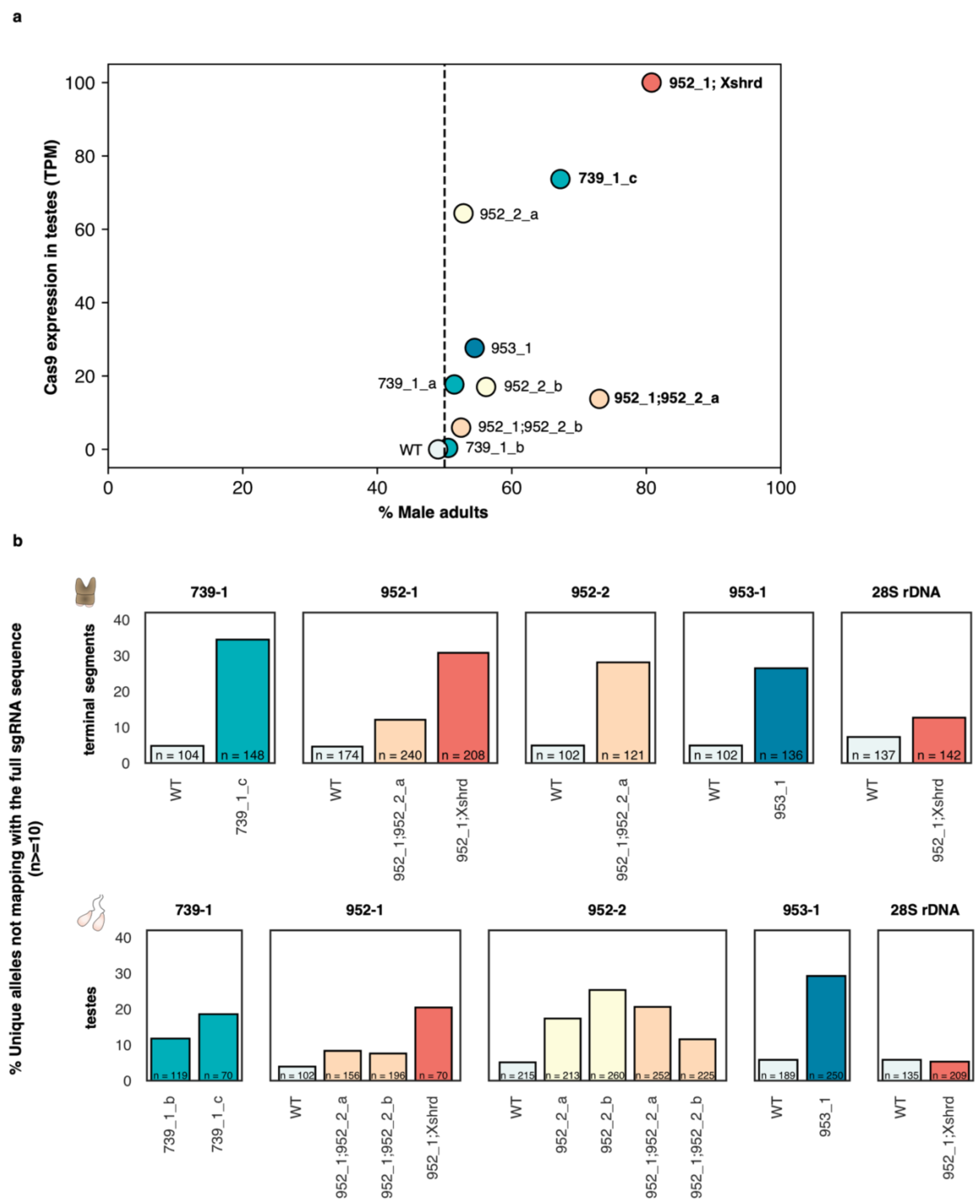

Figure S3. Analysis of CRISPR activity (a) Relationship between Cas9 expression in testes (in Transcripts Per Million, TPM) and induced male bias at the adult stage in offspring of transgenic

males of every X-targeting strain generated in the study and wild type (WT) control. **(b)** Frequency of unique alleles not matching the reference sgRNA target sites from amplicon sequencing of genomic DNA extracted from posterior, terminal abdomens (top) and dissected testes (bottom). “n” indicates the total number of unique alleles occurring at least 10 times in the data. Source data are provided as a Source Data files.

(a) 739-1

Sample	Sequence	Mutation	Frequency
Reference	P A D K K G D V G V G A G		
	CCGGCCGACAAGAAGGGAGACGTCGGTGTCGGTGCCGGCG		
739_1_c Testes (n=64,636)	CCGGCCGACAAG--------CGTCGGTGTCGGTGCCGGCG	Δ8	(0.73%)
	CCGGCCGAC------------GTCGGTGTCGGTGCCGGCG	Δ12	(0.72%)
	CCGGCCGACA----------CGTCGGTGTCGGTGCCGGCG	Δ10	(0.63%)
	CCGGCCGAC-----------CGTCGGTGTCGGTGCCGGCG	Δ11	(0.45%)
	CCGGCCGACAAGAAG-----CGTCGGTGTCGGTGCCGGCG	Δ5	(0.43%)
	CCGGCCGACAAGAA------CGTCGGTGTCGGTGCCGGCG	Δ6,K>N	(0.41%)
	CCGGCCGACAAGA-------CGTCGGTGTCGGTGCCGGCG	Δ7	(0.38%)
	CCGGCCGACAA---------CGTCGGTGTCGGTGCCGGCG	Δ9,K>N	(0.28%)
	CCGGCCGACAAGAAGGGAG-CGTCGGTGTCGGTGCCGGCG	Δ1	(0.15%)
	CCGGCCG-------------CGTCGGTGTCGGTGCCGGCG	Δ13	(0.14%)
739_1_c Surviving females (n=101,824)	CCGGCCGAC------------GTCGGTGTCGGTGCCGGCG	Δ12	(5.17%)
	CCGGCCGAC------------GTCGGGGTCGGTGCCGGCG	Δ12,G>G	(0.07%)
	CCGGCCGAC------------GTCGGTGTCGGGGCCGGCG	Δ12,G>G	(0.03%)
	CCGGCCGAC------------GGCGGTGTCGGTGCCGGCG	Δ12,V>G	(0.03%)
	CCGGCCGAC------------GTCGGTGGCGGTGCCGGCG	Δ12,V>G	(0.02%)
	CCGGCCGAC------------GCCGGTGTCGGTGCCGGCG	Δ12,V>A	(0.02%)
	CCGGCCGGC------------GTCGGTGTCGGTGCCGGCG	D>G,Δ12	(0.02%)
	CCGGCCGACGAGAAGGGAGACGTCGGGGTCGGTGCCGGCG	K>E,G>G	(0.01%)
	CCGGCCGAC------------GTCGGAGTCGGTGCCGGCG	Δ12,G>G	(0.01%)
	CCGGCCGAC------------GTCGGCGTCGGTGCCGGCG	Δ12,G>G	(0.01%)

(b) 952-1

Sample	Sequence	Mutation	Frequency
Reference	K R R R T T G T G R M R Y		
	TAAGCGCAGGAGAACCACCGGTACCGGTCGTATGCGCTAC		
952_1;952_2_a Testes (n=126,800)	TAAGCGCAGGAGAACCACCG------------TGCGCTAC	Δ12,G>V	(0.15%)
	TAAGCGCAGGAGAACCACCG-------------GCGCTAC	Δ13	(0.11%)
	TAAGCGCAGGAGAACCACCG---------------GCTAC	Δ15,G>G	(0.07%)
	TAAGCGCAGGAGAACCACCG-----GGCCGTATGCGCTAC	Δ5	(0.06%)
	TAAGCGCAGGAGAACCACCG--------CGTATGCGCTAC	Δ8	(0.06%)
	TAAGCGCAGGAGAACCACCG-----------ATGCGCTAC	Δ11	(0.03%)
	TAAGCGCAGGAGAACCACCG--ACCGGCCGTATGCGCTAC	Δ2	(0.02%)
	TAAGCGCAGGAGAACCACCG------GCCGTATGCGCTAC	Δ6,G>G	(0.02%)
	TAAGCGCAGGAGAACCACCG-TCCCGGCCGTATGCGCTAC	Δ1	(0.02%)
	TAAGCGCAGGAGAACCCCCG---------------GCTAC	T>P,Δ15,G>G	(0.02%)
952_1;952_2_a Surviving females (n=129,704)	TAAGCGCAGGAGACCCTCCGGTACCGGCCGTATGCGCTCC	T>P,T>U,G>G,Y>S	(0.01%)
	TAAGCGCAGGAGACCCACCGGTACCTGCCGTATGCGCTAC	T>P,G>C	(0.01%)
Xshrd;952_1 Testes (n=118,815)	TAAGCGCAGGAGAACCACCG------GCCGTATGCGCTAC	Δ6,G>G	(0.22%)
	TAAGCGCAGGAGAACCACCG----CGGCCGTATGCGCTAC	Δ4	(0.20%)
	TAAGCGCAGGAGAACCACCG-----GGCCGTATGCGCTAC	Δ5	(0.13%)
	TAAGCGCAGGAGAACCACCG--------CGTATGCGCTAC	Δ8	(0.09%)
	TAAGCGCAGGAGAACCACCG----------TATGCGCTAC	Δ10	(0.09%)
	TAAGCGCAGGAGAACCA---GTACCGGCCGTATGCGCTAC	Δ3,T>S	(0.08%)
	TAAGCGCAGGAGAACCACCG-----------ATGCGCTAC	Δ11	(0.08%)
	TAAGCGCAGGAGAAC-----GTACCGGCCGTATGCGCTAC	Δ5	(0.07%)
	TAAGCGCAGGAGAACCACCG--ACCGGCCGTATGCGCTAC	Δ2	(0.05%)
	TAAGCGCAGGAGAA------GTACCGGCCGTATGCGCTAC	Δ6,T>S	(0.05%)
Xshrd;952_1 Surviving females (n=110,446)	None		

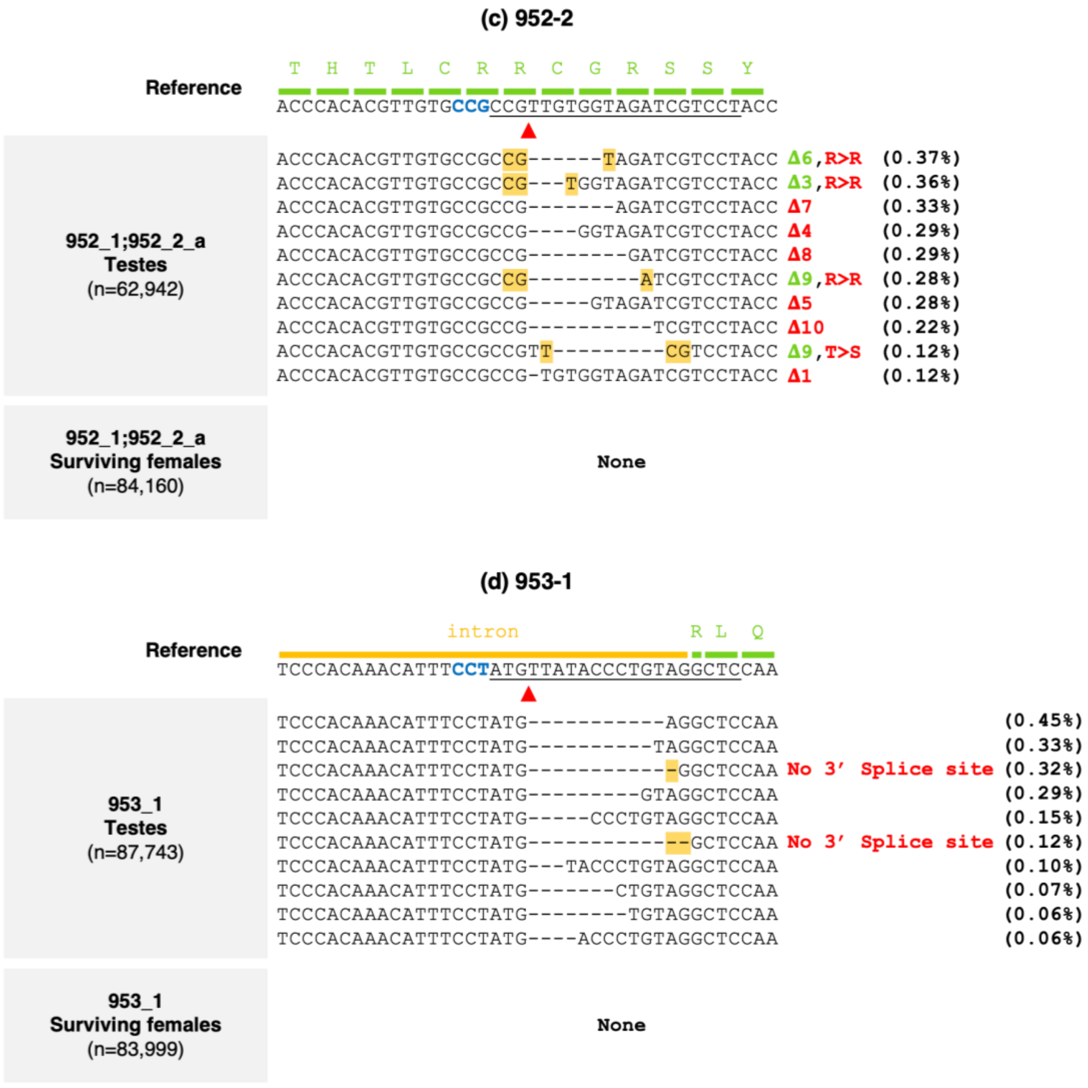

Figure S4. Analysis of CRISPR-induced mutant alleles in testes and surviving females. This figure presents the top 10 most prevalent CRISPR-induced alleles (i.e., alleles which do not perfectly match the reference sgRNA target sites and were absent in wild type samples) in both testes and surviving female progeny of X-targeting strains. The sample type, strain ID, and the reference sequence at the corresponding site, with the amino acid sequence highlighted in green, are shown. The Cas9 cut site is denoted by a red arrow, and the PAM site is highlighted in blue. Deletions are represented by dashes, while substitutions are shown in red. The number of deleted

amino acids is indicated by Δ and the impact on the reading frame in red or green, for out- and in-frame mutations, respectively. Substitutions that result in amino acid replacement are indicated in red while substitutions that do not result in amino acid replacement are indicated in green. “n” represents the total reads for all alleles occurring at least 10 times in the dataset. Additionally, the percentage of reads for each CRISPR-induced allele, relative to the total reads, is provided in brackets. **(a)** 739_1 target site **(b)** 952_1 target site **(c)** 952_1 target site **(d)** 953_1 target site. Source data are provided as a Source Data files.

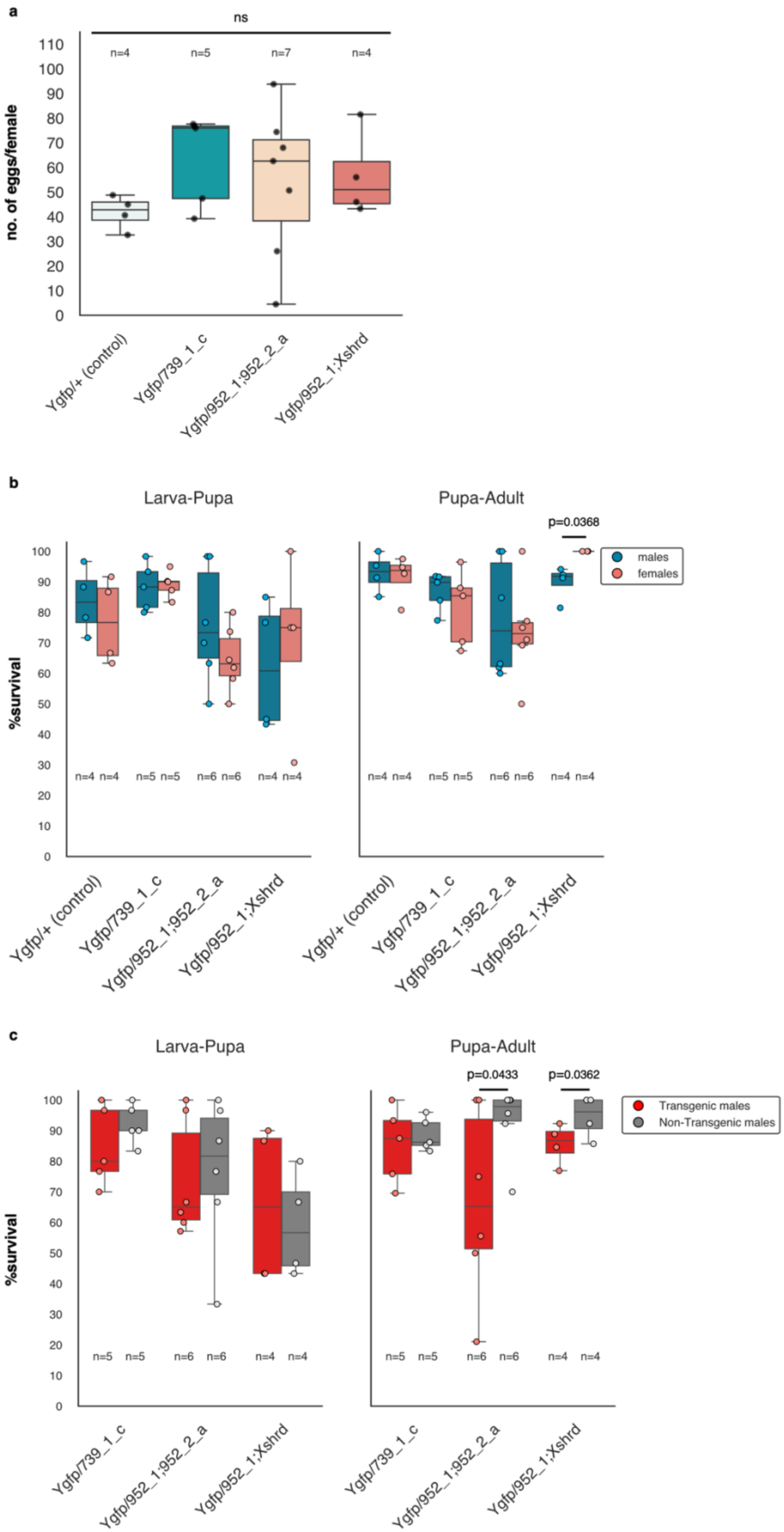
a
ns
no. of eggs/female
110
100
90
80
70
60
50
40
30
20
10
0
n=4
n=5
n=7
n=4
Ygfp/+ (control)
Ygfp/739_1_c
Ygfp/952_1;952_2_a
Ygfp/952_1;Xshrd
b
Larva-Pupa
Pupa-Adult
p=0.0368
males
females
%survival
100
90
80
70
60
50
40
30
20
10
0
n=4 n=4
n=5 n=5
n=6 n=6
n=4 n=4
n=4 n=4
n=5 n=5
n=6 n=6
n=4 n=4
Ygfp/+ (control)
Ygfp/739_1_c
Ygfp/952_1;952_2_a
Ygfp/952_1;Xshrd
Ygfp/+ (control)
Ygfp/739_1_c
Ygfp/952_1;952_2_a
Ygfp/952_1;Xshrd
c
Larva-Pupa
Pupa-Adult
p=0.0433
p=0.0362
Transgenic males
Non-Transgenic males
%survival
100
90
80
70
60
50
40
30
20
10
0
n=5 n=5
n=6 n=6
n=4 n=4
n=5 n=5
n=6 n=6
n=4 n=4
Ygfp/739_1_c
Ygfp/952_1;952_2_a
Ygfp/952_1;Xshrd
Ygfp/739_1_c
Ygfp/952_1;952_2_a
Ygfp/952_1;Xshrd

Figure S5. Supporting data of sex-specific mortality assays in *An. gambiae.* (a) Number of eggs per female. Statistical analysis was done using a One-Way ANOVA ($F(3, 16) = 0.7511$, $p = 0.5376$). Survival rates at pupation (Larva-Pupa) and adult emergence (Pupa-Adult) of **(b)** male (blue) vs. female (coral) F1 progeny and of **(c)** DsRed+ transgenic (red) vs. DsRed- non-transgenic (gray) male F1 progeny. Statistical analysis was done using two-sided paired t-tests, assuming equal or unequal variances, comparing the survival rates of male individuals with that of female individuals and of SRD+ male individuals with those of SRD- male individuals at both pupation and adult emergence stages ($\alpha = 0.05$). "n" indicated the number of biologically independent replicates used to derive statistics. Boxplots show median values (line), the interquartile range (IQR, box), minima & maxima (whiskers). Individual data points represent data from each replicate. Source data are provided as a Source Data files.

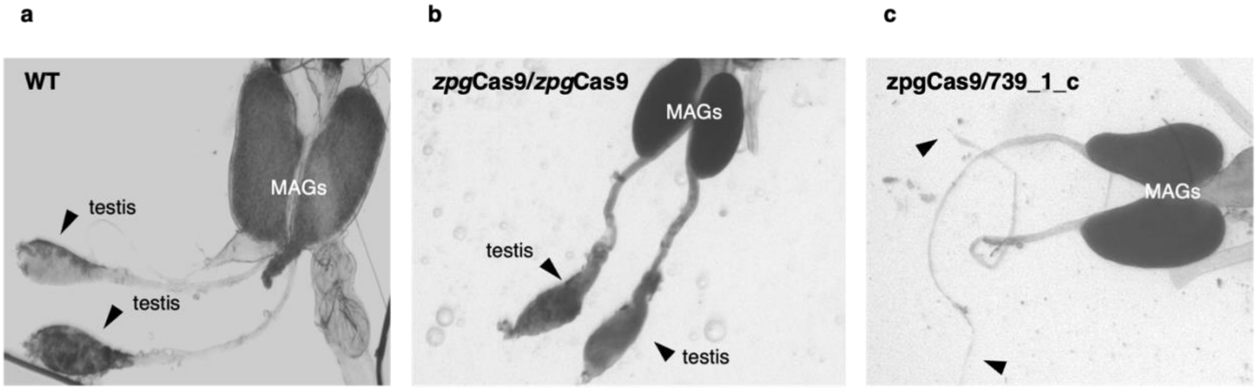

Figure S6. Testes morphology of *zpg*Cas9/739_1_c sterile males and controls. Male reproductive tissues of **(a)** wild type (WT) male **(b)** *zpg*Cas9 homozygous male and (**c**) *zpg*Cas9/739_1_c sterile male. Testes are indicated with a black arrowhead. Male accessory glands are indicated in white (MAGs).

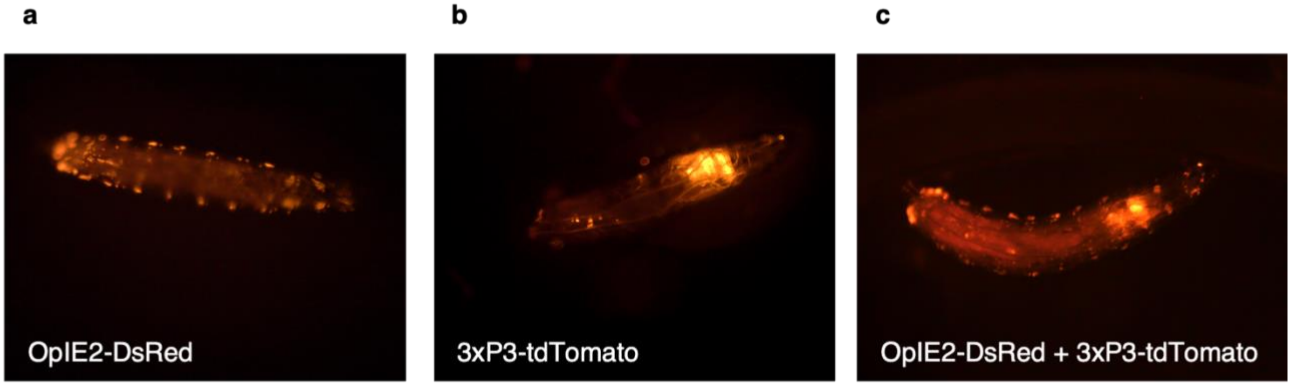

Figure S7. Expression pattern of fluorescent markers in *D. melanogaster* L1 larvae. (a) Autosomal DsRed driven by the OpIE2 promoter **(b)** Y-chromosome linked tdTomato driven by the 3xP3 promoter **(c)** combined expression of autosomal OpIE2-DsRed and Y-linked 3xP3-tdTomato.

Supplementary References

1. Papa, F. et al. Rapid evolution of female-biased genes among four species of *Anopheles* malaria mosquitoes. *Genome Res.* **27,** 1536-1548 (2017).
2. Taxiarchi, C. et al. High-resolution transcriptional profiling of *Anopheles gambiae* spermatogenesis reveals mechanisms of sex chromosome regulation. *Sci. Rep.* **9,** 14841 (2019).
