# Supplementary material for: Targeting mosquito X-chromosomes reveals complex transmission dynamics of sex ratio distorting gene drives
Source: Nat Commun. 2024 Jun 11;15:4983. doi: 10.1038/s41467-024-49387-7 (PMC11166636; doi:10.1038/s41467-024-49387-7)
Supplement: Supplementary file 2 — Reporting Summary [file 41467_2024_49387_MOESM2_ESM.pdf]

nature portfolio

Corresponding author(s): Philippos Aris Papathanos

Last updated by author(s): 05/20/2024

Reporting Summary

Nature Portfolio wishes to improve the reproducibility of the work that we publish. This form provides structure for consistency and transparency in reporting. For further information on Nature Portfolio policies, see our Editorial Policies and the Editorial Policy Checklist.

Statistics

For all statistical analyses, confirm that the following items are present in the figure legend, table legend, main text, or Methods section.

n/a	Confirmed	
[ ]	[x]	The exact sample size (*n*) for each experimental group/condition, given as a discrete number and unit of measurement
[ ]	[x]	A statement on whether measurements were taken from distinct samples or whether the same sample was measured repeatedly
[ ]	[x]	The statistical test(s) used AND whether they are one- or two-sided<br>*Only common tests should be described solely by name; describe more complex techniques in the Methods section.*
[x]	[ ]	A description of all covariates tested
[ ]	[x]	A description of any assumptions or corrections, such as tests of normality and adjustment for multiple comparisons
[ ]	[x]	A full description of the statistical parameters including central tendency (e.g. means) or other basic estimates (e.g. regression coefficient) AND variation (e.g. standard deviation) or associated estimates of uncertainty (e.g. confidence intervals)
[ ]	[x]	For null hypothesis testing, the test statistic (e.g. *F*, *t*, *r*) with confidence intervals, effect sizes, degrees of freedom and *P* value noted<br>*Give P values as exact values whenever suitable.*
[x]	[ ]	For Bayesian analysis, information on the choice of priors and Markov chain Monte Carlo settings
[x]	[ ]	For hierarchical and complex designs, identification of the appropriate level for tests and full reporting of outcomes
[x]	[ ]	Estimates of effect sizes (e.g. Cohen's *d*, Pearson's *r*), indicating how they were calculated

Our web collection on statistics for biologists contains articles on many of the points above.

Software and code

Policy information about availability of computer code

Data collection	No software was used to collect data in the study.
Data analysis	Statistical data analysis was performed using JMP Pro 15 software. RNA-seq data were analyzed by mapping reads to the Anopheles gambiae genome (Vectorbase, A. gambiae genome, version AgamP64) using HiSat2, and normalized to Transcripts Per Million (TPM). Graphic representations (figures) were prepared using Python and Microsoft® PowerPoint Version 16.84.1 (24042223).

For manuscripts utilizing custom algorithms or software that are central to the research but not yet described in published literature, software must be made available to editors and reviewers. We strongly encourage code deposition in a community repository (e.g. GitHub). See the Nature Portfolio guidelines for submitting code & software for further information.

Data

Policy information about availability of data

All manuscripts must include a data availability statement. This statement should provide the following information, where applicable:

- Accession codes, unique identifiers, or web links for publicly available datasets
- A description of any restrictions on data availability
- For clinical datasets or third party data, please ensure that the statement adheres to our policy

The authors declare that the data supporting the findings of this study are available within the paper and its supplementary information files. Raw input data, scripts used to generate the figure panels presented in the paper, and statistical analysis files are available on figshare with the identifier https://doi.org/10.6084/m9.figshare.c.704679835. Sequencing data generated by RNA-seq and amplicon sequencing analyses have been deposited in the NIH's Sequence Read Archive

(SRA) with the accession code PRJNA1070274 (https://www.ncbi.nlm.nih.gov/bioproject/?term=PRJNA1070274). Source data for all relevant figures are provided with the paper.

Research involving human participants, their data, or biological material

Policy information about studies with human participants or human data. See also policy information about sex, gender (identity/presentation), and sexual orientation and race, ethnicity and racism.

Reporting on sex and gender	N/A
Reporting on race, ethnicity, or other socially relevant groupings	N/A
Population characteristics	N/A
Recruitment	N/A
Ethics oversight	N/A

Note that full information on the approval of the study protocol must also be provided in the manuscript.

Field-specific reporting

Please select the one below that is the best fit for your research. If you are not sure, read the appropriate sections before making your selection.

[x] Life sciences [ ] Behavioural & social sciences [ ] Ecological, evolutionary & environmental sciences

For a reference copy of the document with all sections, see nature.com/documents/nr-reporting-summary-flat.pdf

Life sciences study design

All studies must disclose on these points even when the disclosure is negative.

Sample size	The number of biological replicates used to derive statistics for each experiment is described in within each figure, and is consistent with previous literature (//doi.org/10.1371/journal.pgen.1008647) (n > 3). No statistical method was used to predetermine sample size. Since our study deals with insect populations, we were able to use a very large sample size within individual replicates, strengthening our conclusions for each experiment. The total number of individuals used for every experiment is also provided in the article within each figure.
Data exclusions	No data were excluded from the raw data appearing in the Source Data files. If specific data points were excluded from downstream statistical analyses (such as in the PCR-based single embryo genotyping), this is clearly mentioned in the relevant methods section and appears in the Source Data file.
Replication	The number of biological replicates is provided within each figure and in the relevant method section, as well as in raw data tables. Each experimental cross was repeated a minimum of 3 times.
Randomization	All experimental crosses were set up using randomly sampled individuals within each selected transgenic or wild type group.
Blinding	No formal blinding was conducted since our data is quantitative and not subjective. However, whenever possible, we employed a form of blinding in which the experimenter analyzing the results (counting, sexing, or assessing phenotype) was not aware of the identity (i.e., strain or cross) of each replicate. This approach reduces experimenter bias.

Reporting for specific materials, systems and methods

We require information from authors about some types of materials, experimental systems and methods used in many studies. Here, indicate whether each material, system or method listed is relevant to your study. If you are not sure if a list item applies to your research, read the appropriate section before selecting a response.

Materials & experimental systems

n/a	Involved in the study	
[x]	[ ]	Antibodies
[x]	[ ]	Eukaryotic cell lines
[x]	[ ]	Palaeontology and archaeology
[ ]	[x]	Animals and other organisms
[x]	[ ]	Clinical data
[x]	[ ]	Dual use research of concern
[x]	[ ]	Plants

Methods

n/a	Involved in the study	
[x]	[ ]	ChIP-seq
[x]	[ ]	Flow cytometry
[x]	[ ]	MRI-based neuroimaging

Animals and other research organisms

Policy information about studies involving animals; ARRIVE guidelines recommended for reporting animal research, and Sex and Gender in Research

Laboratory animals	Anopheles gambiae G3 strain: To establish crosses, mosquitoes were counted, sexed (if relevant), and placed in the cages as pupae, where they emerged as adults and were allowed to mate for 4-7 days prior to blood feeding. Fluorescent screening was performed on L1/L2 larvae or pupae.<br><br>Drosophila melanogaster: The strains used were w[1118] stock, a transgenic gRNA line carrying attP-3B (BDSC #9750), an autosomal β-tubulin Cas-9 line carrying attP40 (BDSC #25709), and the transgenic Y-linked AByG line (BDSC #78567). Crosses were performed with flies 1-4 days old.
Wild animals	The study did not involve wild animals.
Reporting on sex	All sex-based analyses were indicate in manuscript .
Field-collected samples	The study did not involve sample collected from the field.
Ethics oversight	ARO Institutional Animal Care and Use Committee approval number 2307-118-2-VOL-IL.

Note that full information on the approval of the study protocol must also be provided in the manuscript.

Plants

Seed stocks	*Report on the source of all seed stocks or other plant material used. If applicable, state the seed stock centre and catalogue number. If plant specimens were collected from the field, describe the collection location, date and sampling procedures.*
Novel plant genotypes	*Describe the methods by which all novel plant genotypes were produced. This includes those generated by transgenic approaches, gene editing, chemical/radiation-based mutagenesis and hybridization. For transgenic lines, describe the transformation method, the number of independent lines analyzed and the generation upon which experiments were performed. For gene-edited lines, describe the editor used, the endogenous sequence targeted for editing, the targeting guide RNA sequence (if applicable) and how the editor was applied.*
Authentication	*Describe any authentication procedures for each seed stock used or novel genotype generated. Describe any experiments used to assess the effect of a mutation and, where applicable, how potential secondary effects (e.g. second site T-DNA insertions, mosiacism, off-target gene editing) were examined.*
